# Supplementary material for: Gene expression of functionally-related genes coevolves across fungal species: detecting coevolution of gene expression using phylogenetic comparative methods
Source: BMC Genomics. 2020 May 20;21:370. doi: 10.1186/s12864-020-6761-3 (PMC7240986; doi:10.1186/s12864-020-6761-3)
Supplement: Supplementary file 1 — Additional file 1Supplemental Material and Methods. Files contains supplemental materials and methods, including supplementarytables and figures referenced in this manuscript. Table S1. Species and data sources used in this study. Basic information on species used in analysis, including the citation corresponding to the RNA-Seq data used and whether or not STRING data was available at the time of analysis. Table S2. Comparing performance of different methods for assessing coevolution of gene expression after removing protein pairs with a STRING Score less than 400. Figure S1. Heatmap showing overall similarity between species gene expression estimates. Figure S2. Strength of coevolution as function of overlapping Gene Ontology terms between protein pairs. Figure S3. Effects of controlling for number of interactions of proteins on phylogenetically-corrected and phylogenetically-uncorrected correlations. Figure S4. Distance between CAI and empirical-based measures of coevolution as function of gene expression. Figure S5. Results from simulated data. Figure S6. Phylogenetically-corrected correlation distributions without filtering genes violating BM. Figure S7. Effects of functional-relatedness on phylogenetically corrected correlation without filtering genes violating BM. Figure S8. Effects of number of interactions and gene expression on strength of coevolution without filtering genes violating BM. Figure S9. Comparison to other coevolution metrics based on protein sequence and CAI without filtering genes violating BM. Figure S10. Assessing the effects of potential discordance between the species tree and gene trees (e.g. introgression, ILS, etc.). Figure S11. Assessing the effects of using branch lengths based on mean nucleotides substitutions per site (as output by RAxML) instead of a time-calibrated tree. [file 12864_2020_6761_MOESM1_ESM.pdf]

Supplemental Information: Gene Expression of Functionally-Related Genes Co-evolves Across Fungal Species

Alexander L. Cope[1,4], Brian O'Meara[1,2,3], and Michael A. Gilchrist[1,2,3]

[1]Genome Science and Technology, University of Tennessee, Knoxville
[2]Department of Ecology and Evolutionary Biology, University of Tennessee,Knoxville
[3]National Institute for Mathematical and Biological Synthesis, Knoxville, TN
[4]Chemical Sciences Division, Oak Ridge National Laboratory, Oak Ridge, TN

Friday 24th April, 2020

Supplemental Methods

Species	Phylum	Family	Sequence Accession (Citation)	STRING Data Available
Saccharomyces cerevisiae	Ascomycota	Saccharomycetaceae	SRA417121 [1]	Yes
Saccharomyces paradoxus	Ascomycota	Saccharomycetaceae	SRA423931 [2]	No
Saccharomyces mikatae	Ascomycota	Saccharomycetaceae	SRA423931 [2]	No
Saccharomyces bayanus	Ascomycota	Saccharomycetaceae	SRA246981 [3]	No
Saccharomyces kudriavzevii	Ascomycota	Saccharomycetaceae	SRA246981 [3]	No
Lachancea kluyverii	Ascomycota	Saccharomycetaceae	ERA489180 [4]	Yes
Naumovozyma castellii	Ascomycota	Saccharomycetaceae	SRA246981 [3]	No
Candida glabrata	Ascomycota	Saccharomycetaceae	SRA185486 [5]	Yes
Candida albicans	Ascomycota	Debaryomycetaceae	SRA756982 [6]	Yes
Candida parapsilosis	Ascomycota	Debaryomycetaceae	SRA645737 [7]	Yes
Fusarium graminearum	Ascomycota	Nectriaceae	SRA436010 [8]	Yes
Magnaporthe oryzae	Ascomycota	Magnaporthaceae	SRA107966 [9]	Yes
Aspergillus fumigatus	Ascomycota	Trichocomaceae	SRA551938 [10]	No
Aspergillus nidulans	Ascomycota	Trichocomaceae	SRA742708 [11]	Yes
Neurospora crassa	Ascomycota	Sordariaceae	SRA059445 [12, 13, 14]	Yes
Neurospora discreta	Ascomycota	Sordariaceae	SRA178585 [12, 13, 14]	No
Neurospora tetrasperma	Ascomycota	Sordariaceae	SRA178586 [12, 13, 14]	No
Cryotpococcus neoformans	Basidiomycota	Tremellaceae	SRA417121 [1]	No

Table S1: Basic information on species used in analysis, including the citation corresponding to the RNA-Seq data used and whether or not STRING data was available at the time of analysis.

Method	Correlation	TPR (S.D)	FPR (S.D)	FDR (S.D)	Accuracy (S.D)
PCM	ρ_C	0.568 (0.0003)	0.031 (0.0037)	0.051 (0.0056)	0.769 (0.0018)
cor.test()	ρ_U	0.640 (0.0011)	0.216 (0.0102)	0.252 (0.0087)	0.712 (0.00346)
Fraser et. al.	ρ_C	0.629 (0.0531)	0.041 (0.0195)	0.060 (0.0234)	0.794 (0.0189)
	ρ_U	0.578 (0.0426)	0.086 (0.0293)	0.128 (0.0314)	0.746 (0.0162)
Martin and Fraser	ρ_C	0.580 (0.0121)	0.031 (0.0009)	0.050 (0.0009)	0.775 (0.0057)
	ρ_U	0.390 (0.0195)	0.021 (0.0013)	0.050 (0.0015)	0.685 (0.0092)

Table S2: Performance metrics for 18 species fungal tree using simulated data for detecting coevolution. By excluding potential false positives from the simulated binding group (i.e. STRING Score < 400), the overall accuracy of the methods improves, but the approaches based on the phylogenetically-corrected correlation ρ_C remain superior.

Quantifying functional-relatedness via Gene Ontology terms

One might imagine proteins which have more overlapping GO terms are involved in more of the same functional processes, and thus would show stronger coevolution of gene expression. To quantify functional-relatedness via GO terms, the Jaccard index was used. Briefly, or a protein pair with GO terms A and B, the Jaccard Index is defined as

$$\text{Jaccard Index} = \frac{|A \cap B|}{|A \cup B|}$$

Simulations

All simulations were carried out using **mvMORPH**. Data were simulated from the binding group allowing evolutionary covariance term Cov_E to be non-zero and simulated data from control group forcing $\text{Cov}_E = 0$. The binding group was simulated using the corresponding MLEs of the evolutionary rate matrix and ancestral state estimates from the real data. The control group was simulated similarly, but the evolutionary covariance Cov_E parameter was fixed to be 0.0 (i.e. independent evolution of gene expression). Simulations used standard error estimates from the real data.

Supplemental Figures

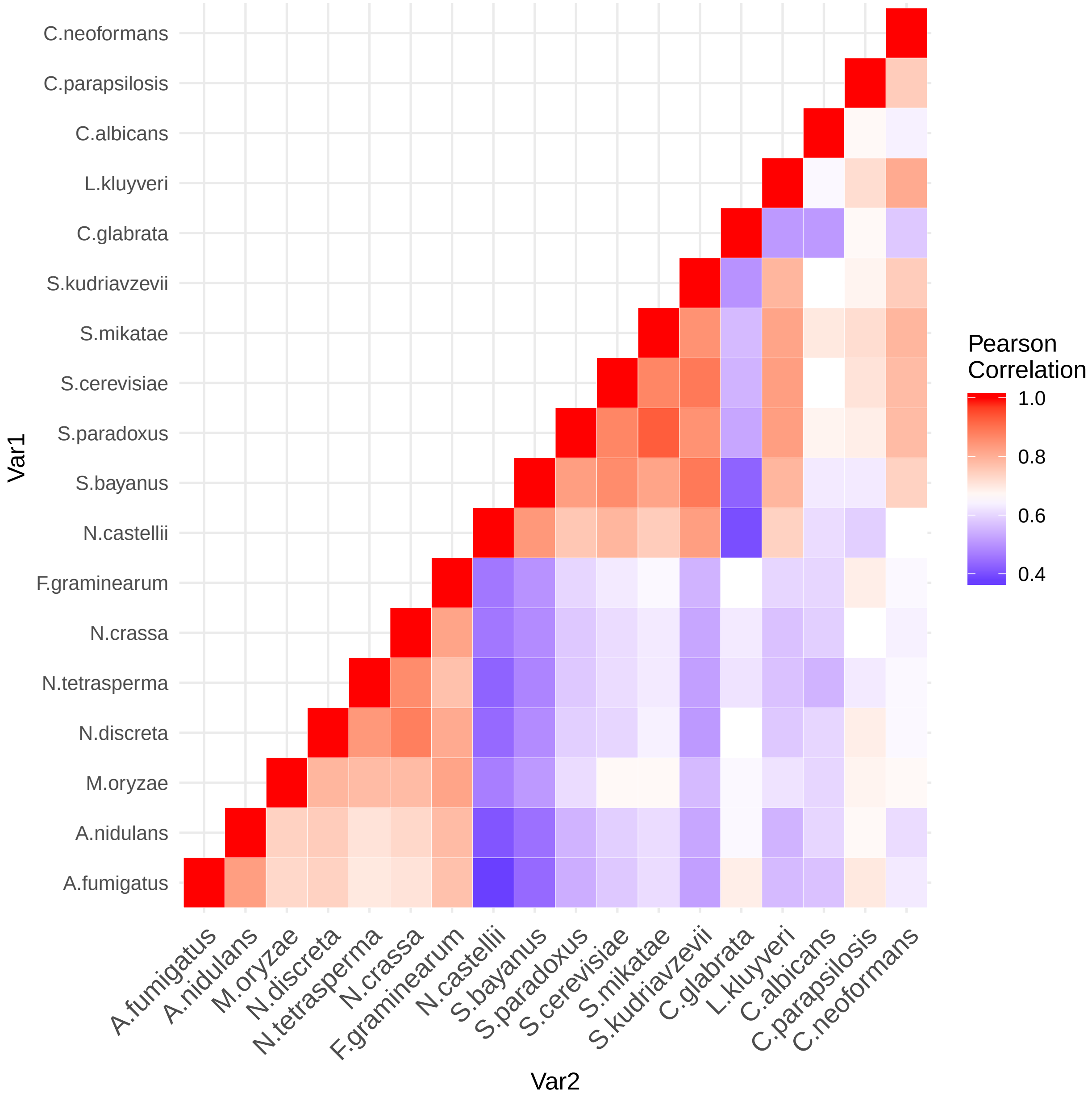

Figure S1: Heatmap demonstrating the correlation between normalized gene expression values of the 18 fungal species. Species which are more closely related tend to show higher correlations in overall gene expression patterns. *Candida* species appear to be exceptions, although gene expression is still moderately correlated with the other *Saccharomycotina* species.

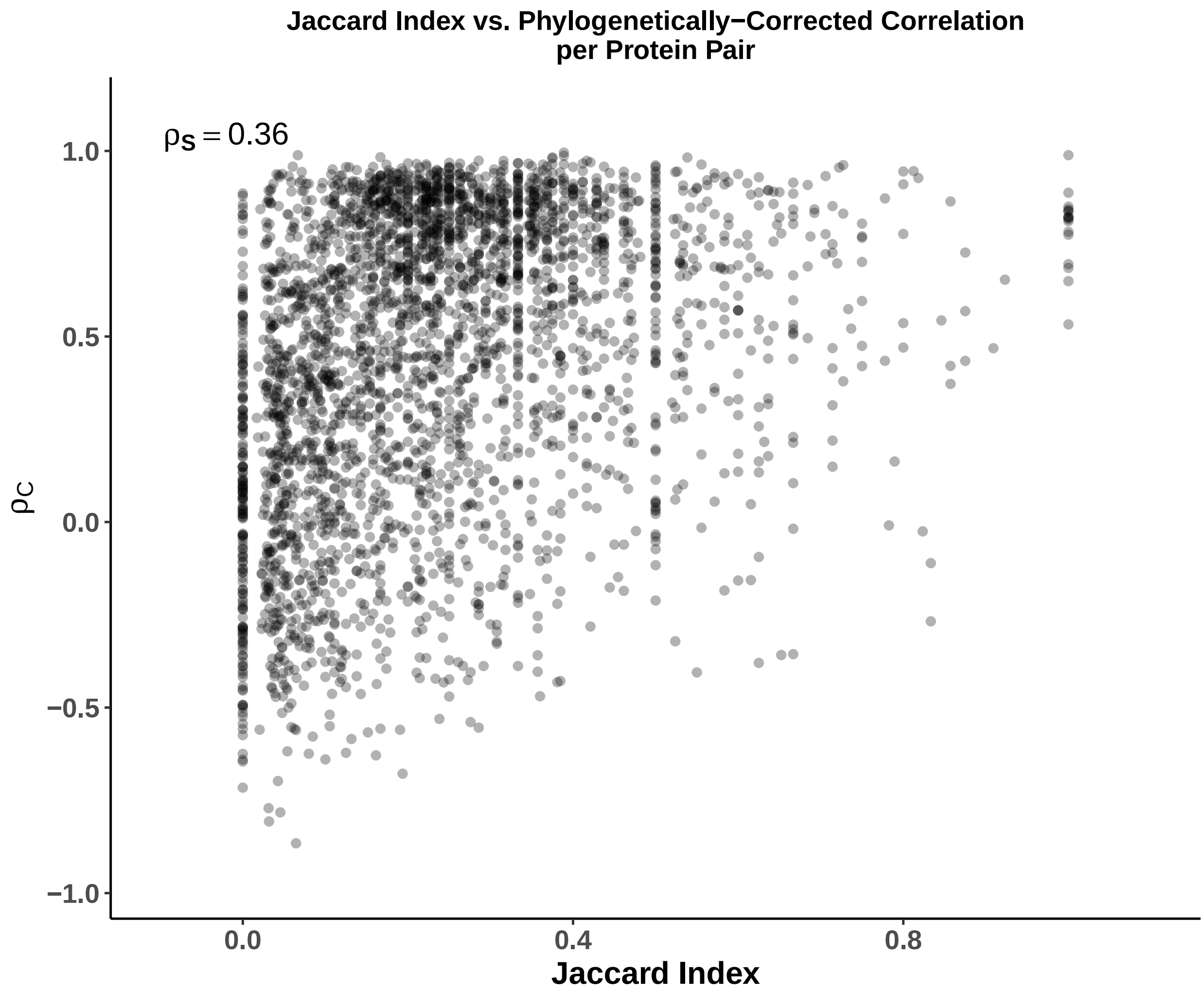

Figure S2: Pairs of proteins with more overlapping GO terms tend to show stronger coevolution of gene expresssion (Weighted Spearman Rank Correlation $\rho_S = 0.36$, 95% CI: 0.306 – 0.408, $p < 10^{-41}$). The Jaccard Index reflects functional similarity between two proteins based on GO terms.

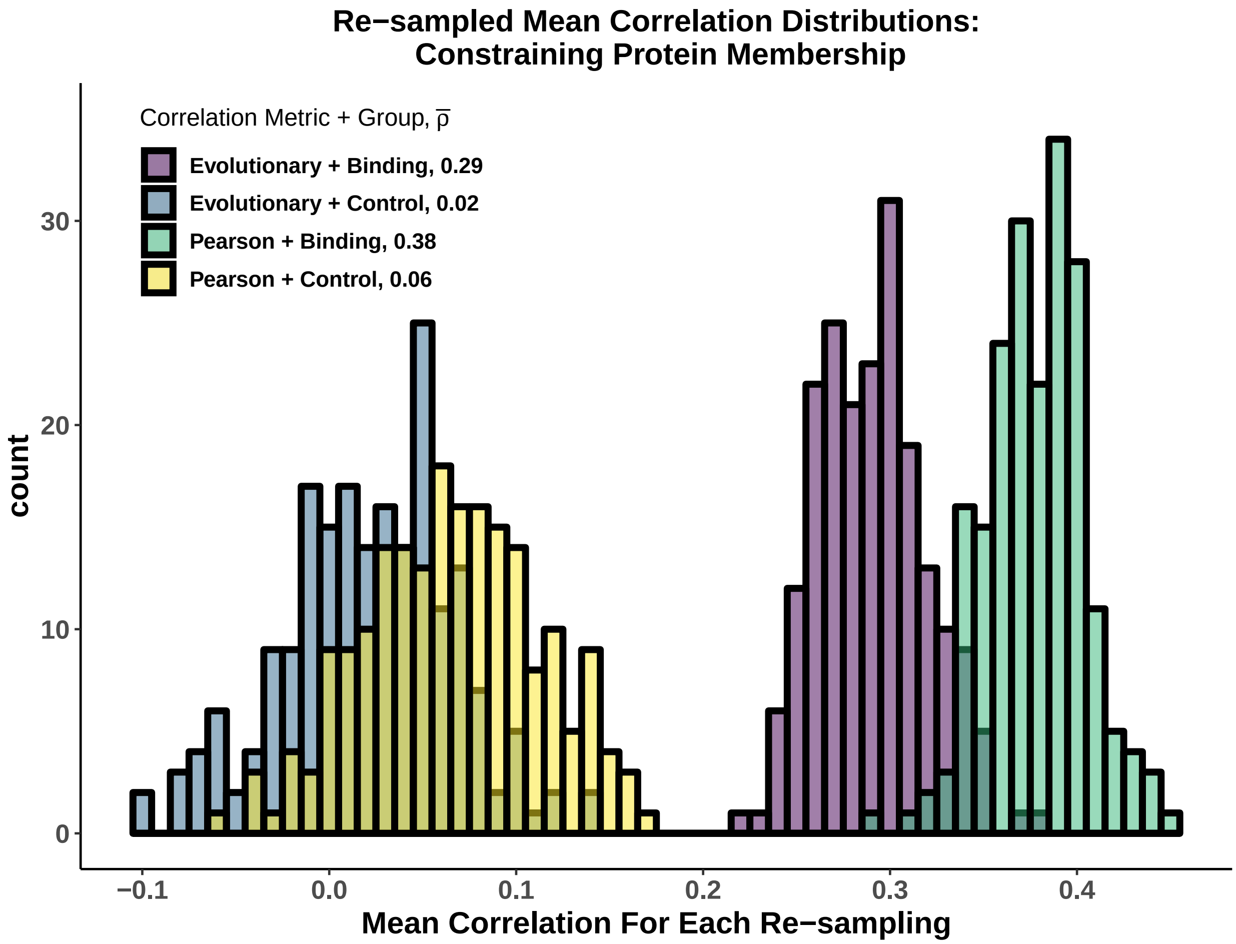

Figure S3: Distributions reflect mean phylogenetically-corrected $\bar{\rho}_C$ and phylogenetically-uncorrected $\bar{\rho}_U$ estimates for each of the 200 re-samplings of the binding and control datasets, in which each protein is restricted to being in only one pair per dataset, at max. Results are mostly consistent with results not restricting protein membership, although there does appear to be less discrepancy between the binding and control groups.

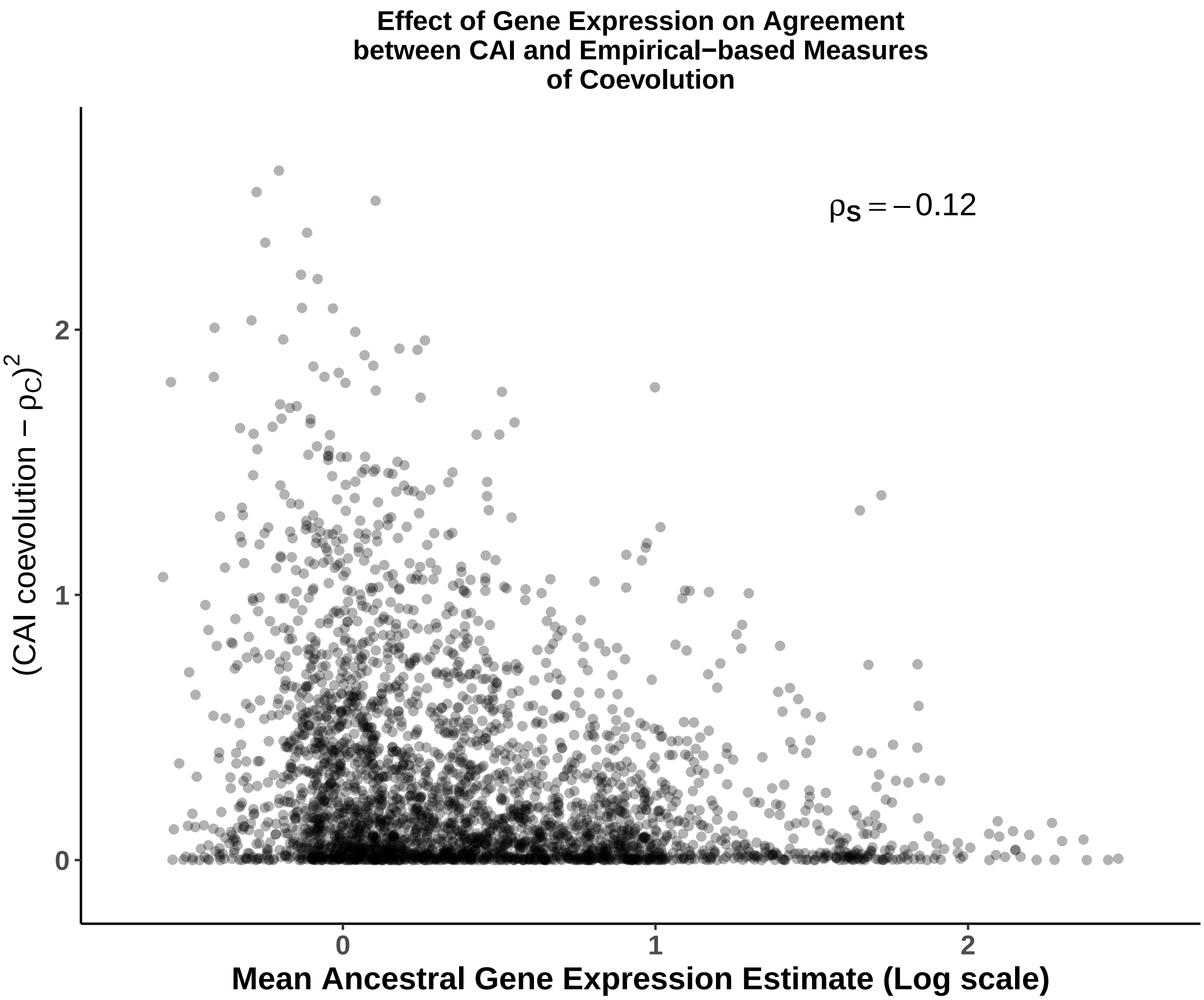

Figure S4: Determining the impact of gene expression on the agreement between CAI coevolution and ρ_C, as measured by the squared difference between the two metrics. A negative correlation (Weigthed Spearman Rank $\rho_S = -0.12$, $p < 10^{-4}$), indicates protein pairs which are, on average, more highly expressed tend to show less discrepancy between the CAI and empirical-based measures of gene expression coevolution.

Results without filtering genes violating BM assumption

The analysis was repeated not excluding genes which violated the BM assumption. This does appear to reduce some of the weighted Spearman rank correlations, but the overall conclusions are consistent with the more conservative dataset.

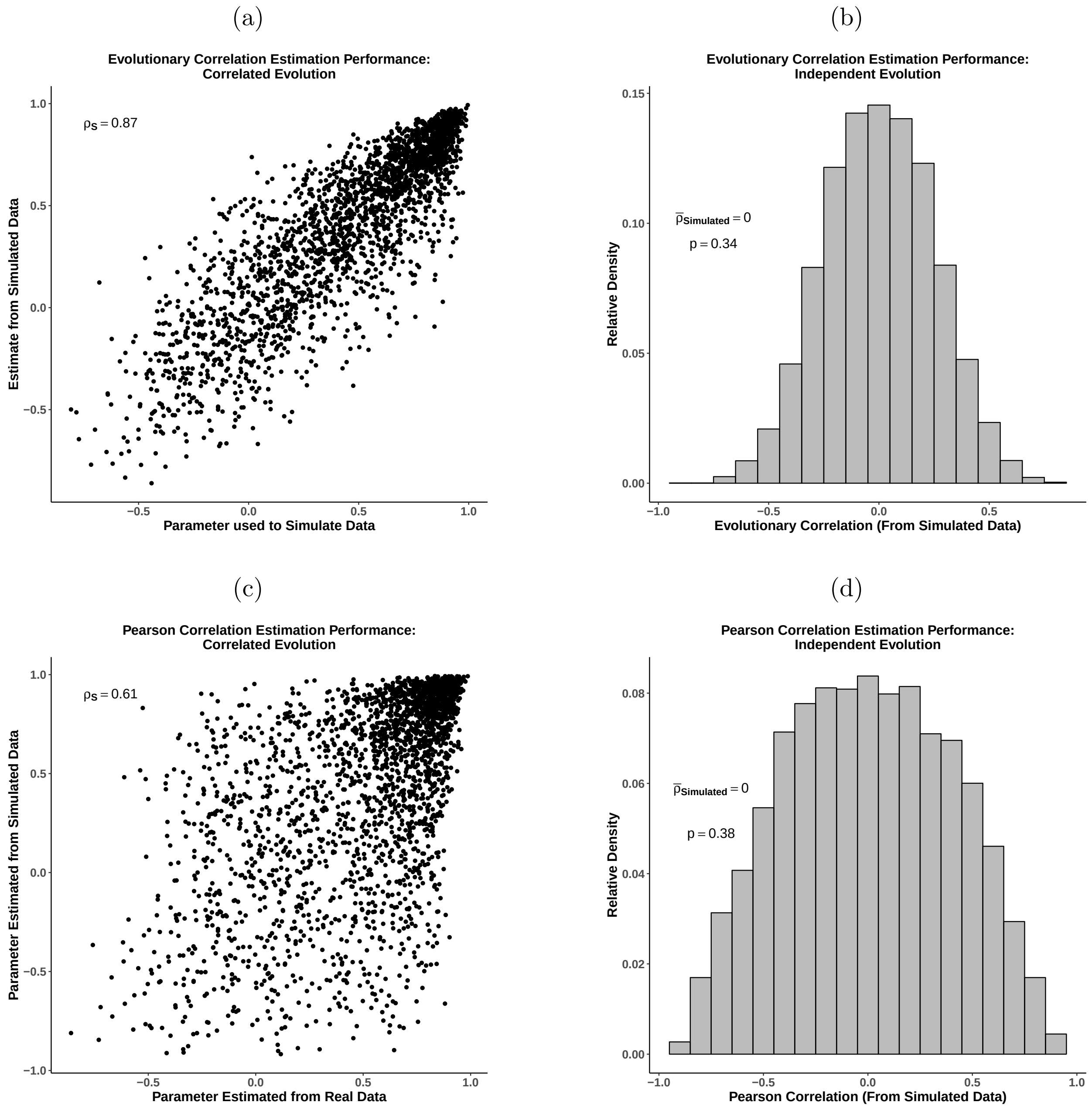

Figure S5: Estimates of phylogenetically-corrected correlation ρ_P and phylogenetically-uncorrected correlation ρ_U from Brownian Motion simulations when phylogenetically-corrected correlation rho_C is (a,c) allowed to vary from 0.0 and (b,d) is restricted to be 0.0 (i.e. independent evolution of gene expression). (a) Comparison of ρ_C from simulated data to the MLEs of ρ_C used to simulate data allowing for coevolution of gene expression. Estimates from simulated data are strongly correlated with MLE from real data. (b) Distribution of ρ_C estimated from simulated data forcing independent evolution of gene expression (i.e. $\rho_E = 0.0$ for all protein pairs in data set). Distribution is centered around 0.0, as expected under the null (One-Sample t-test, $p = 0.34$). (c,d) Same as (a) and (b), but for ρ_U. Results deviate much more from expectation when not accounting for phylogeny.

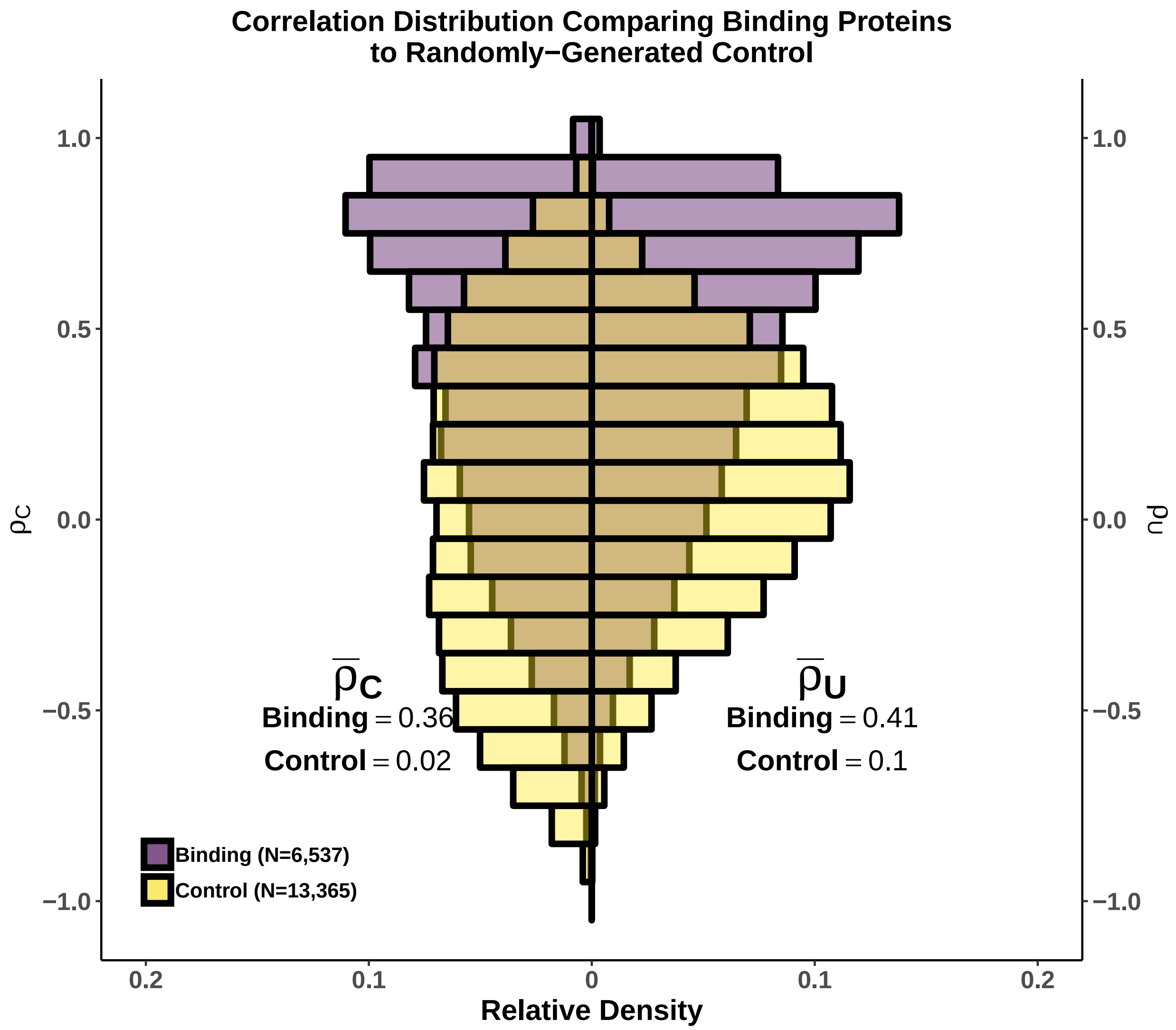

Figure S6: Comparing the distributions of the phylogenetically-corrected correlation ρ_C and phylogenetically-uncorrected correlation ρ_U for the binding (purple) and control (yellow) groups without filtering genes which violate the BM assumption. Mean values for the binding and control group phylogenetically-corrected correlation ρ_C distributions are 0.36 ($p < 10^{-200}$) and 0.02 ($p < 10^{-8}$), respectively. Mean values for the binding and control group phylogenetically-uncorrected correlation ρ_U distributions are 0.41 ($p < 10^{-300}$) and 0.10 ($p < 10^{-300}$), respectively.

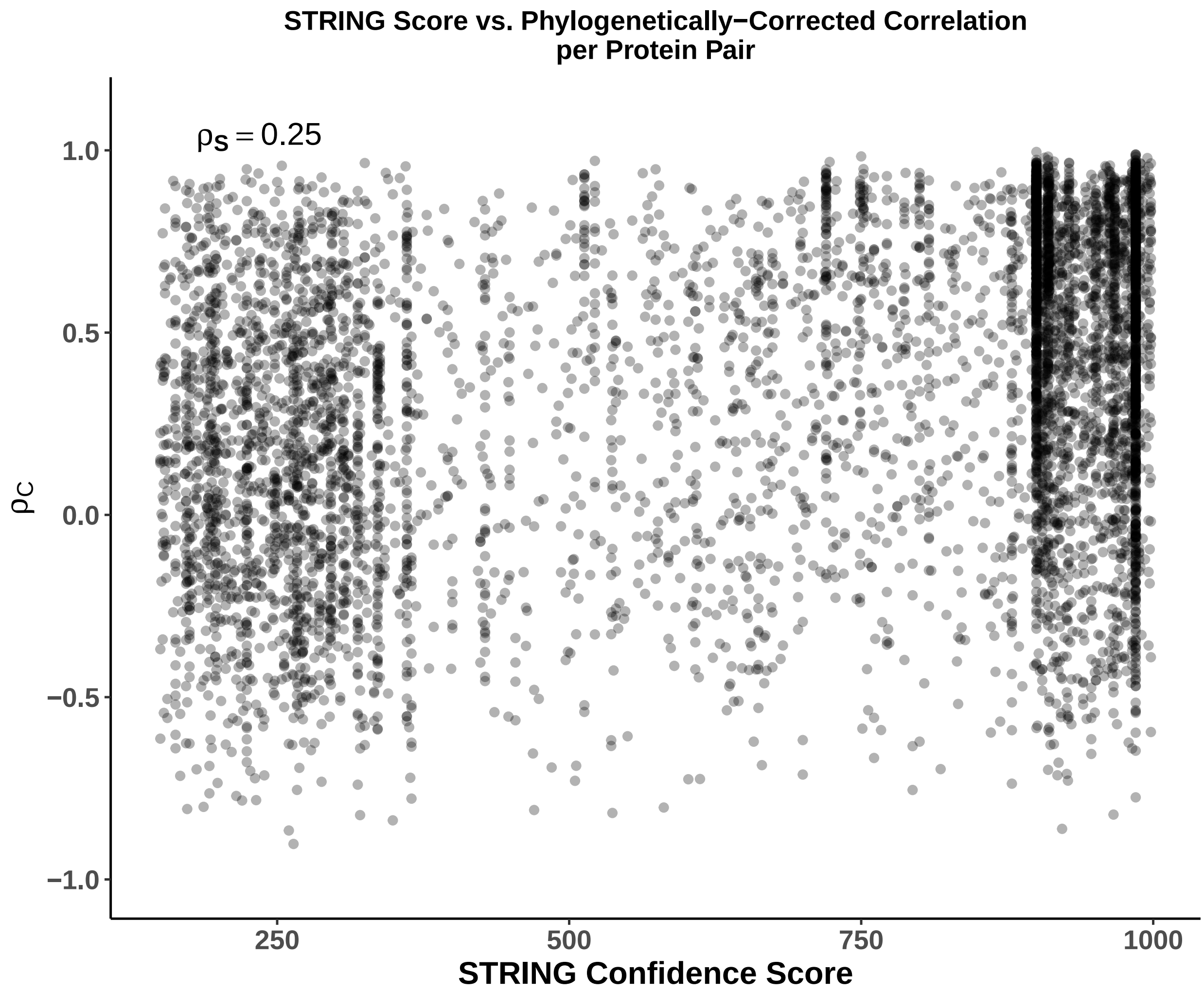

Figure S7: Effects of metric representing functional-relatedness on phylogenetically-corrected correlation ρ_C. Positive weighted Spearman rank correlation ($\rho_S = 0.25$, $p < 10^-48$) between the STRING score and phylogenetically-corrected correlation ρ_C indicates more confident and/or conserved interactions tend to have higher ρ_C, indicating stronger coevolution at the gene expression level.

(a) (b)

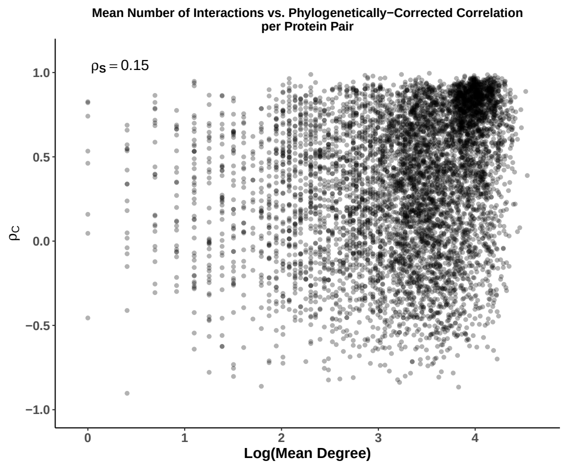

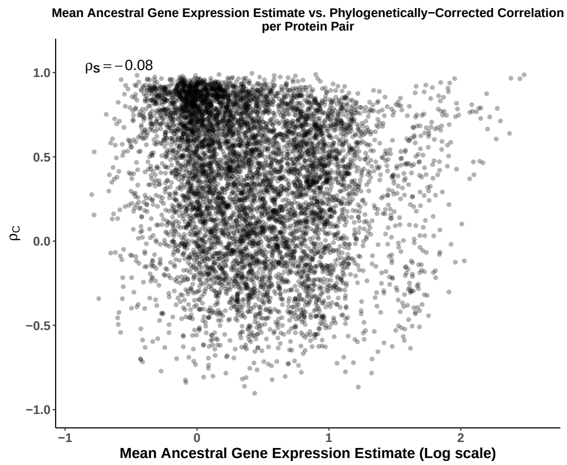

Figure S8: The relationship of (a) the mean degree (average number of interactions between a protein pair) and (b) mean ancestral gene expression estimate with the phylogenetically-corrected correlation ρ_C for the binding group. Both protein pair metrics are weakly, but significantly correlated with the phylogenetically-corrected correlation ρ_C: weighted Spearman rank correlation $\rho_S = 0.15$ ($p < 10^{-10}$) for mean degree and $\rho_S = -0.08$ ($p < 10^{-4}$) for mean ancestral gene expression. This suggests both metrics are poor predictors of the strength of coevolution of gene expression between protein pairs.

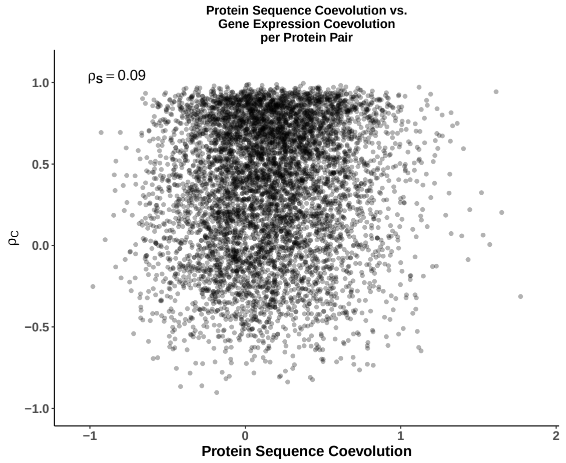

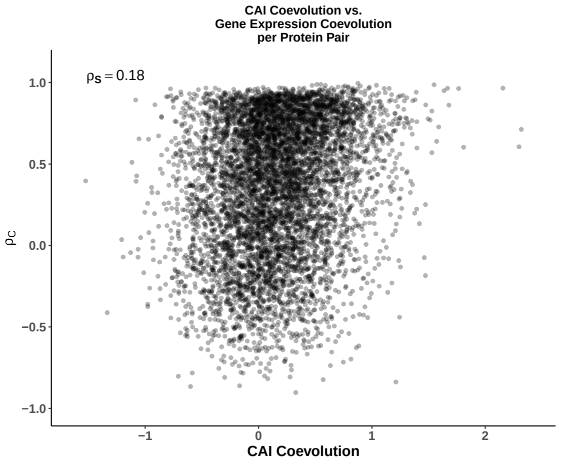

Figure S9: (a) Comparing coevolution of gene expression, represented by the phylogenetically-corrected correlation ρ_C, and protein sequences, as described in the main text. There is a weak but significant correlation (Weighted Spearman Rank Correlation $\rho_S = 0.09$, $p < 10^{-4}$) between the measures of gene expressions and protein sequence coevolution. (b) A similar comparison using the measures of CAI coevolution as described in main manuscript. Again, there is a weak, but significant correlation (Weighted Spearman Rank correlation $\rho_S = 0.18$, $p < 10^{-18}$).

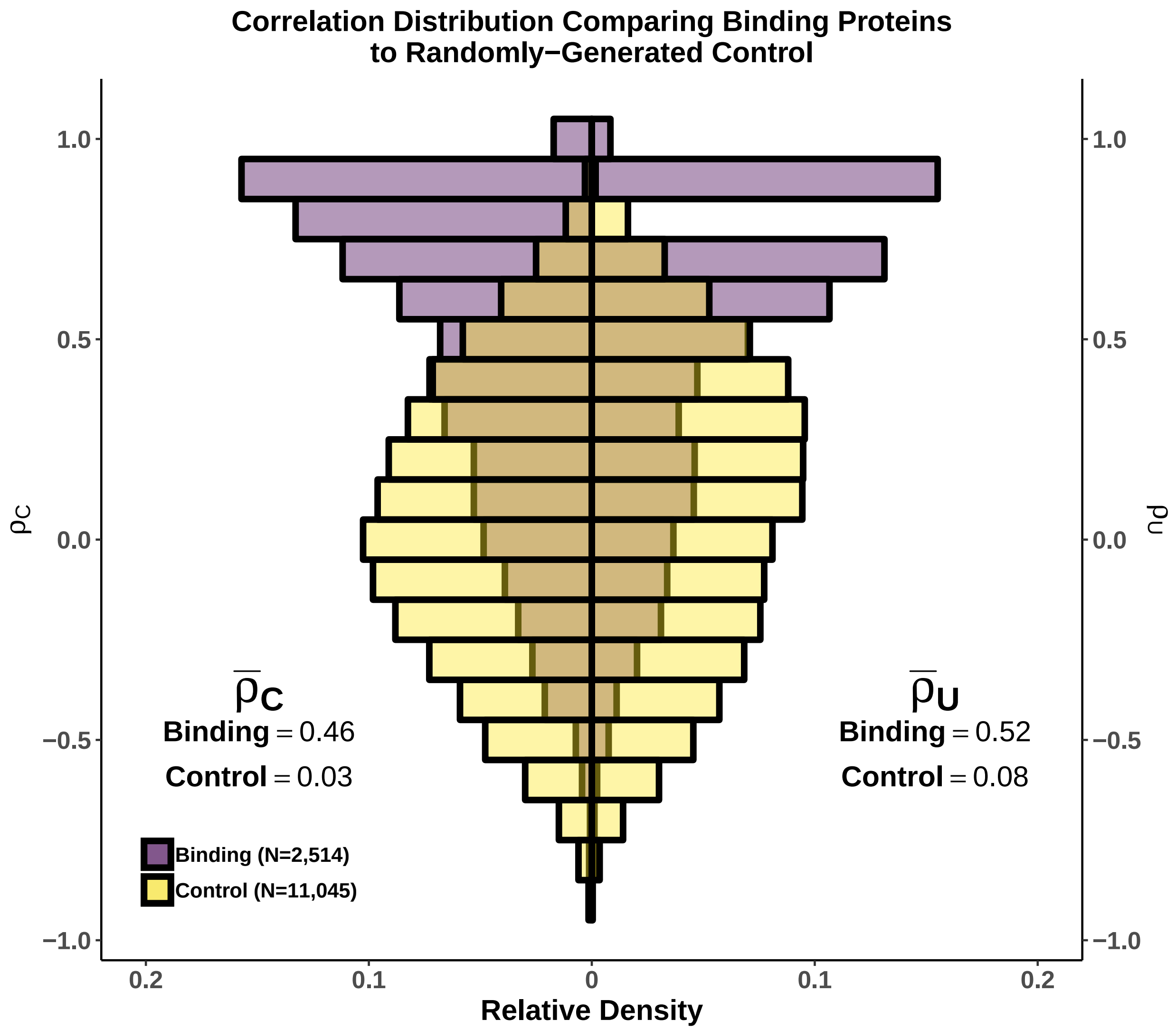

Figure S10: Comparing the distributions of the phylogenetically-corrected correlation ρ_C and phylogenetically-uncorrected correlation ρ_U for the binding (purple) and control (yellow) groups filtering out possible introgression and horizontal gene transfer genes from the analysis.

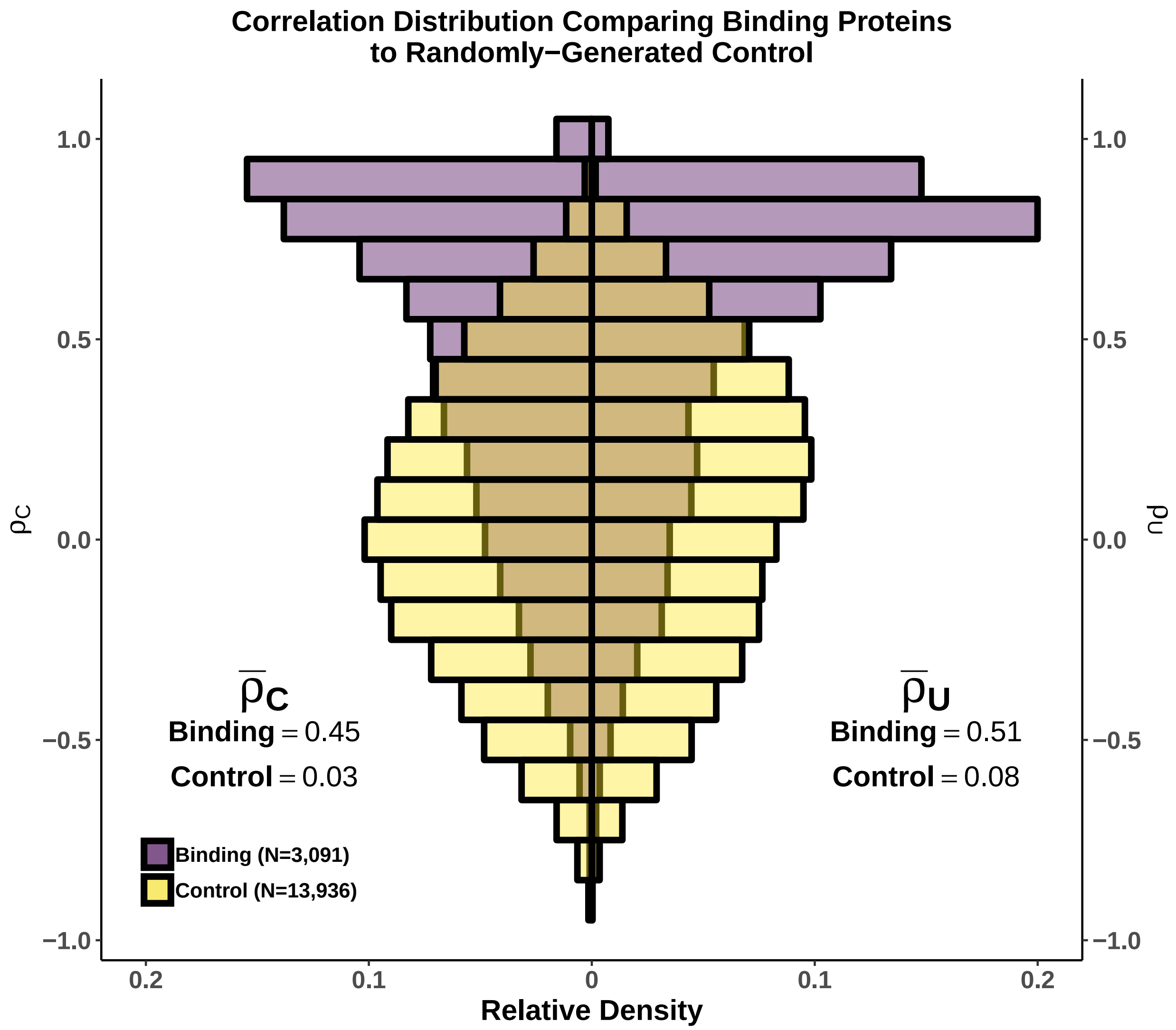

Figure S11: Comparing the distributions of the phylogenetically-corrected correlation ρ_C and phylogenetically-uncorrected correlation ρ_U for the binding (purple) and control (yellow) groups using the tree output by RaxML. This tree has branch lengths in units of mean nucleotide substitutions per site.

References

[1] Kelliher CM, Leman AR, Sierra CS, Haase SB. Investigating Conservation of the Cell-Cycle-Regulated Transcriptional Program in the Fungal Pathogen, Cryptococcus neoformans. PLoS Genet. 2016 dec;12(12).

[2] Yang JR, Maclean CJ, Park C, Zhao H, Zhang J. Intra and interspecific variations of gene expression levels in yeast are largely neutral: (Nei Lecture, SMBE 2016, Gold Coast). Mol Biol Evol. 2017;34(9):2125–2139.

[3] Alcid EA, Tsukiyama T. Expansion of antisense lncRNA transcriptomes in budding yeast species since the loss of RNAi. Nat Struct Mol Biol. 2016 may;23(5):450–455.

[4] Brion C, Pflieger D, Souali-Crespo S, Friedrich A, Schacherer J. Differences in environmental stress response among yeasts is consistent with species-specific lifestyles. Mol Biol Cell. 2016 may;27(10):1694–1705.

[5] Linde J, Duggan S, Weber M, Horn F, Sieber P, Hellwig D, et al. Defining the transcriptomic landscape of Candida glabrata by RNA-Seq. Nucleic Acids Res. 2015;43(3):1392–1406.

[6] del Olmo Toledo V, Puccinelli R, Fordyce PM, Pérez JC. Diversification of DNA binding specificities enabled SREBP transcription regulators to expand the repertoire of cellular functions that they govern in fungi. PLoS Genet. 2018 dec;14(12).

[7] Turner SA, Ma Q, Ola M, Martinez de San Vicente K, Butler G. Dal81 Regulates Expression of Arginine Metabolism Genes in Candida parapsilosis . mSphere. 2018 mar;3(2).

[8] Puri KD, Yan C, Leng Y, Zhong S. RNA-seq revealed differences in transcriptomes between 3ADON and 15ADON populations of Fusarium graminearum in vitro and in planta. PLoS One. 2016 oct;11(10).

[9] Choi J, Chung H, Lee GW, Koh SK, Chae SK, Lee YH. Genome-wide analysis of hypoxia-responsive genes in the rice blast fungus, Magnaporthe oryzae. PLoS One. 2015 aug;10(8).

[10] Manfiolli AO, de Castro PA, dos Reis TF, Dolan S, Doyle S, Jones G, et al. Aspergillus fumigatus protein phosphatase PpzA is involved in iron assimilation, secondary metabolite production, and virulence. Cell Microbiol. 2017 dec;19(12).

[11] Pidroni A, Faber B, Brosch G, Bauer I, Graessle S. A class 1 histone deacetylase as major regulator of secondary metabolite production in Aspergillus nidulans. Front Microbiol. 2018 sep;9(SEP).

[12] Wang Z, Kin K, López-Giráldez F, Johannesson H, Townsend JP. Sex-specific gene expression during asexual development of Neurospora crassa. Fungal Genet Biol. 2012 jul;49(7):533–543.

[13] Wang Z, Lopez-Giraldez F, Lehr N, Farré M, Common R, Trail F, et al. Global gene expression and focused knockout analysis reveals genes associated with fungal fruiting body development in Neurospora crassa. Eukaryot Cell. 2014 jan;13(1):154–169.

[14] Lehr NA, Wang Z, Li N, Hewitt DA, López-Giráldez F, Trail F, et al. Gene expression differences among three Neurospora species reveal genes required for sexual reproduction in Neurospora crassa. PLoS One. 2014 oct;9(10).
